# Sources and Sinks of Serine in Nutrition, Health, and Disease

**DOI:** 10.1146/annurev-nutr-061021-022648

**Published:** 2023-06-12

**Authors:** Michal K. Handzlik, Christian M. Metallo

**Affiliations:** Molecular and Cell Biology Laboratory, Salk Institute for Biological Studies, La Jolla, California, USA; Molecular and Cell Biology Laboratory, Salk Institute for Biological Studies, La Jolla, California, USA

**Keywords:** serine, glycine, metabolic flux, neuropathy, diabetes, metabolomics

## Abstract

Amino acid dysregulation has emerged as an important driver of disease progression in various contexts. l-Serine lies at a central node of metabolism, linking carbohydrate metabolism, transamination, glycine, and folate-mediated one-carbon metabolism to protein synthesis and various downstream bioenergetic and biosynthetic pathways. l-Serine is produced locally in the brain but is sourced predominantly from glycine and one-carbon metabolism in peripheral tissues via liver and kidney metabolism. Compromised regulation or activity of l-serine synthesis and disposal occurs in the context of genetic diseases as well as chronic disease states, leading to low circulating l-serine levels and pathogenesis in the nervous system, retina, heart, and aging muscle. Dietary interventions in preclinical models modulate sensory neuropathy, retinopathy, tumor growth, and muscle regeneration. A serine tolerance test may provide a quantitative readout of l-serine homeostasis that identifies patients who may be susceptible to neuropathy or responsive to therapy.

## 1. INTRODUCTION

Nutrition and metabolism are intertwined, with specific biochemicals absorbed, transported, and metabolized through distinct enzymatic pathways. Given the interconnected nature of biochemical pathways, defective import or metabolism of a given nutrient or cofactor may have diverse metabolic and functional consequences on organs throughout the body. This phenomenon is particularly relevant for amino acids, which are the primary subunits of all proteins but also act as precursors for nucleotides, lipids, cofactors, and posttranslational modifications. As such, changes in the intake, absorption, synthesis, and/or catabolism of amino acids may disrupt numerous biochemical processes across diverse tissues to drive different pathologies.

l-Serine is a nonessential α-amino acid (NEAA) important for a variety of biochemical reactions that are critical for the function of many cell types, including proliferating cells (normal and transformed), neurons, stem cells, myocytes, and immune cells. Furthermore, altered metabolic fluxes associated with l-serine metabolism have emerged as potential risk factors for various disease states such as cancer, metabolic syndrome, and associated comorbidities. Importantly, there is a growing therapeutic potential in targeting l-serine homeostasis to influence the onset and progression of outcomes in clinical trials and preclinical models, particularly in the case of neurological disorders. In this article, we review the biochemical properties of l-serine, highlight key nodes of transport and organ-specific physiology, and discuss potential approaches for intervention and diagnostics. Though broad by necessity, this review provides a road map to researchers interested in understanding l-serine physiology and its impact on downstream metabolic processes.

## 2. L-SERINE BIOCHEMISTRY AND MOLECULAR METABOLISM

### 2.1. Chemical Properties of L-Serine and Nutritional Content

**Figure 1:**
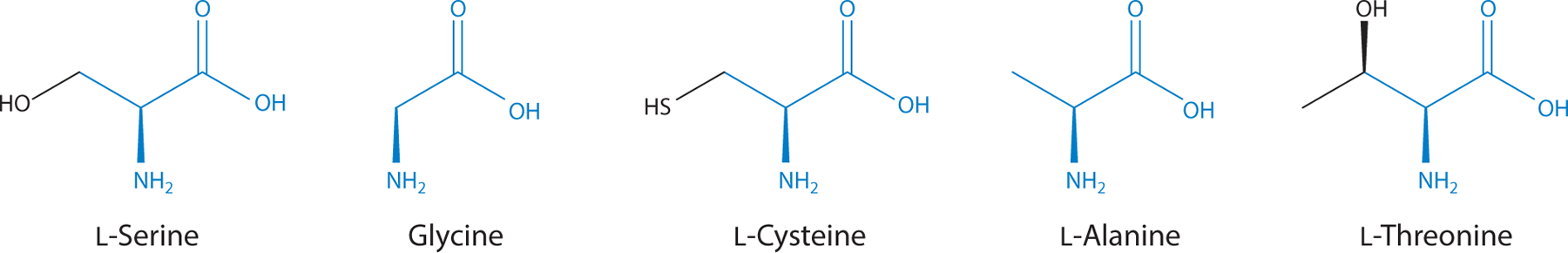
Skeletal formula of l-serine and related amino acids. l-Serine consists of α-amino, carboxyl, and hydroxymethyl groups. The hydroxymethyl group is involved in the transfer of one-carbon units onto tetrahydrofolate for subsequent reactions. Structurally, l-serine is similar to other essential and nonessential amino acids, including glycine, l-cysteine, l-alanine, and l-threonine, thus leading to enzyme promiscuity in the event of aberrant l-serine availability.

l-Serine or serine (symbol Ser or S; molecular mass = 105.093 g/mol) contains an α-amino group, a carboxyl group, and a side chain consisting of a hydroxymethyl group. Under physiological conditions of pH ∼ 7.4, the α-amino group and carboxyl group are protonated and deprotonated, respectively, classifying l-serine as a polar amino acid. Notably, the hydroxymethyl group is used to provide methyl groups for the biosynthesis of amino acids, thymidine, purines, pyrimidines, and pantothenate, thus linking l-serine with numerous metabolic pathways (105). Structurally and metabolically, l-serine is related to glycine, l-cysteine, l-alanine, and l-threonine (Figure 1). As highlighted below, structural similarity renders these species readily interconvertible via distinct enzymes but also promotes the potential for enzyme promiscuity, resulting in atypical reactions in extreme conditions and sometimes driving disease pathologies (see the sidebar titled Enzyme Promiscuity in Amino Acid Metabolism and Its Relation to Disease).

l-Serine was first isolated in 1865 by Emil Cramer (17) from silk protein; hence its name derived from the Latin for silk, *sericum*. l-Serine is abundant in soybeans, nuts (especially peanuts, almonds, and walnuts), eggs, chickpeas, lentils, meat, and fish (especially shellfish). Use of quantitative mass spectrometry revealed that the content of l-serine is 2.2–4.2 g per 100 g in oat, whey, milk, and caseinate (40). Considering the US Department of Agriculture–recommended protein intake for a healthy, sedentary, 50-year-old male individual who weighs 65 kg, an estimated daily l-serine intake likely varies between 1 and 2.5 g/day. In addition to dietary intake, l-serine can be synthesized de novo from carbohydrates, regenerated from glycine and one-carbon units, or recycled from protein breakdown. For this reason, clinical trials quantifying plasma amino acid concentration in healthy individuals abstaining from consumption of meat products found little evidence of systemic l-serine deficiency (22, 101).

Stable isotope tracer infusions in mice showed that steady-state l-serine turnover in circulation is the fourth highest among all amino acids at ∼19 μmol/(kg·min) (51). On the other hand, l-serine turnover in humans was estimated to range from 2.3 to 4.5 μmol/(kg·min) (20, 42), indicating that rodents have increased l-serine turnover relative to humans. To put these values in perspective, in humans, whole-body l-serine turnover equals 0.2–0.3% that of glucose [whole-body glucose turnover rate in humans was estimated to be ∼11–13 mmol/(kg·min)], whereas in mice it approximates 13% that of glucose (51, 112, 118). Finally, urinary excretion of amino acids including l-serine is relatively low in healthy subjects (18), implying that l-serine homeostasis is maintained primarily via dietary intake and/or its catabolism.

### 2.2. L-Serine Biosynthesis from Carbohydrates

**Figure 2:**
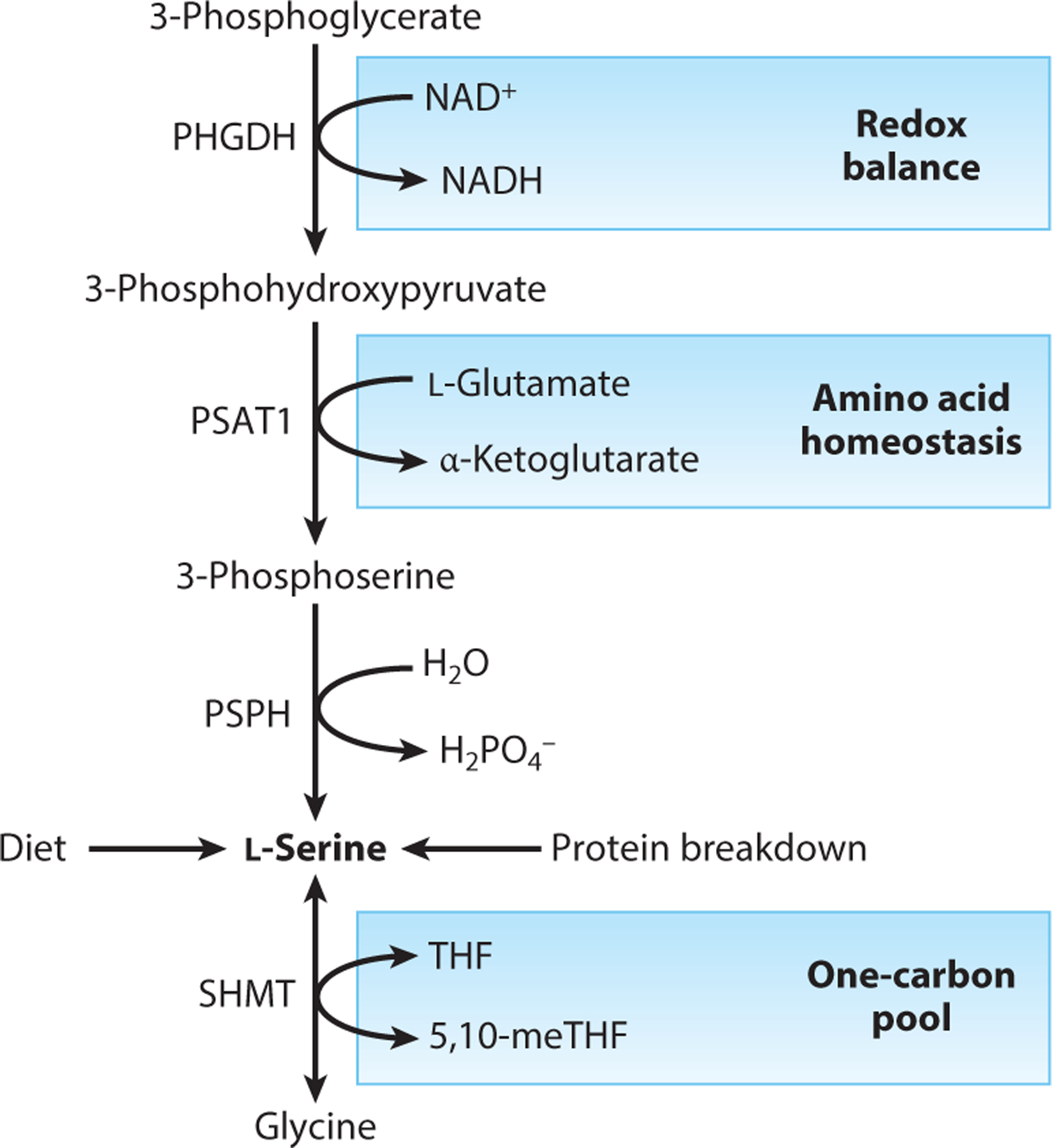
Major metabolic sources of l-serine. De novo l-serine biosynthesis from glucose is catalyzed by three enzymes, including PHGDH, PSAT1, and PSPH. Regeneration from 5,10-methylene tetrahydrofolate (5,10-meTHF) and glycine also occurs in selected tissues. Notably, l-serine biosynthesis is linked to redox balance, amino acid homeostasis via l-glutamate availability, and 5,10-meTHF availability; thus, one-carbon metabolism serves as both a source and a sink for serine. Alterations in these coupled metabolic pathways may therefore affect l-serine synthesis from glucose and glycine. Abbreviations: PHGDH, 3-phosphoglycerate dehydrogenase; PSAT1, 3-phosphoserine aminotransferase 1; PSPH, phosphoserine phosphatase; SHMT, serine hydroxymethyltransferase; 5,10-meTHF, 5,10-methylene tetrahydrofolate.

De novo l-serine synthesis from glucose is initiated by conversion of the glycolytic intermediate 3-phosphoglycerate into 3-phosphohydroxypyruvate in an NAD-dependent reaction catalyzed by 3-phosphoglycerate dehydrogenase (PHGDH) (Figure 2). Phosphoserine aminotransferase 1 (PSAT1) and phosphoserine phosphatase (PSPH) catalyze subsequent reactions that yield 3-phosphoserine and l-serine, respectively. In vitro tracing experiments with stable isotope tracers in rapidly proliferating cells have shown extensive labeling of l-serine from [U-^13^C]glucose, indicating its fast biosynthetic rates in cancer cells (65). In addition to tumor cells, de novo l-serine biosynthesis is highly active in astrocytes (60), macrophages (96), and epidermal stem cells (6), suggesting that, at least under in vitro conditions, a wide variety of cell types are capable of synthesizing l-serine from glucose.

Intracellular l-serine biosynthesis is coupled with redox state, l-glutamate availability as a source of α-amino group, and folate-mediated one-carbon metabolism (FOCM), highlighting the complex relationships between these metabolic pathways in the maintenance of l-serine homeostasis. For instance, inhibiting Complex I of the mitochondrial electron transport chain reduces de novo l-serine biosynthesis from glucose in cancer cell lines, providing experimental evidence that links l-serine synthesis to mitochondrial function and NAD^+^/NADH balance (21, 79). In addition, de novo l-serine synthesis from glucose is coupled to l-glutamate availability and α-ketoglutarate, and this relationship has been hypothesized to influence tumorigenesis upon genomic PHGDH amplification (93). Expression of PHGDH is highly sensitive to exogenous l-serine availability in cultured cells (69), but the mechanisms underlying this transcriptional response have yet to be elucidated.

### 2.3. Relationship with Glycine and Folate-Mediated One-Carbon Metabolism

l-Serine is also produced from glycine, a closely related NEAA, in a reaction catalyzed by serine hydroxymethyltransferase (SHMT) that uses 5,10-methylene tetrahydrofolate as a cosubstrate (49, 104), establishing a critical link between l-serine and the FOCM pathway. l-Serine and glycine are rapidly exchanged in reactions catalyzed by two protein isoforms of SHMT located in the cytosol (SHMT1) and mitochondria (SHMT2). Cell fractionation and sucrose gradient analyses of Chinese hamster cells demonstrated that the activity of SHMT2 is ∼20 times higher than that of SHMT1 (12). In addition, cells harboring a mutation that inactivates SHMT2 are auxotrophic for glycine (12), implying that SHMT1 cannot fully compensate for a lack of the mitochondrial isoform. Indeed, tracer studies using isotope-labeled serine have demonstrated that one-carbon units used for thymidylate synthesis are generated predominantly via cytosolic SHMT1 (1, 2, 46), and this evidence for directionality within the mitochondria and cytosol has been further supported by the use of compartment-specific mutant isocitrate dehydrogenase reporters of NADPH labeling from [2,3,3-^2^H]serine tracers (63). While transcript expression of SHMT2 is ubiquitous across tissues, SHMT1 expression dominates in the liver and kidney (39), which are key physiological sites of l-serine, glycine, and FOCM (as detailed in Sections 4.1 and 4.2). Notably, these tissues contain significantly more glycine than l-serine, with molar ratios in mouse liver and kidney of 6.6 and 18.6, respectively (44).

The observation of l-serine synthesis from glycine is based on experiments involving fetal ovine hepatocytes, in which supplementation with folates stimulated l-serine biosynthesis (82). As discussed below, synthesis of l-serine from glucose or glycine appears to be cell and tissue specific and may correlate with SHMT isozyme expression or function of the glycine cleavage system, which can supply one-carbon units for l-serine regeneration or other pathways. In addition to glycine, dietary folates and betaine can serve as donors of one-carbon units (24). Betaine infusion into mice demonstrated rapid labeling of l-methionine and *S*-adenosylmethionine, particularly in the liver, indicating tissue-specific utilization of one-carbon groups for homocysteine remethylation (38). Furthermore, mice genetically engineered to be deficient in betaine-homocysteine *S*-methyltransferase, an enzyme that utilizes betaine to remethylate homocysteine to methionine, exhibited markedly elevated levels of homocysteine and visible hepatic tumors, suggesting that betaine plays a central role in hepatic FOCM (109). In support of this notion, betaine supplementation to cystathionine B synthase (CBS)–deficient patients with elevated and reduced plasma levels of homocysteine and l-serine, respectively, normalized abundances of both amino acids (25), supporting the model in which betaine may provide one-carbon units to synthesize l-serine and remethylate homocysteine.

### 2.4. Metabolic Fates of L-Serine in Human Cells

**Figure 3:**
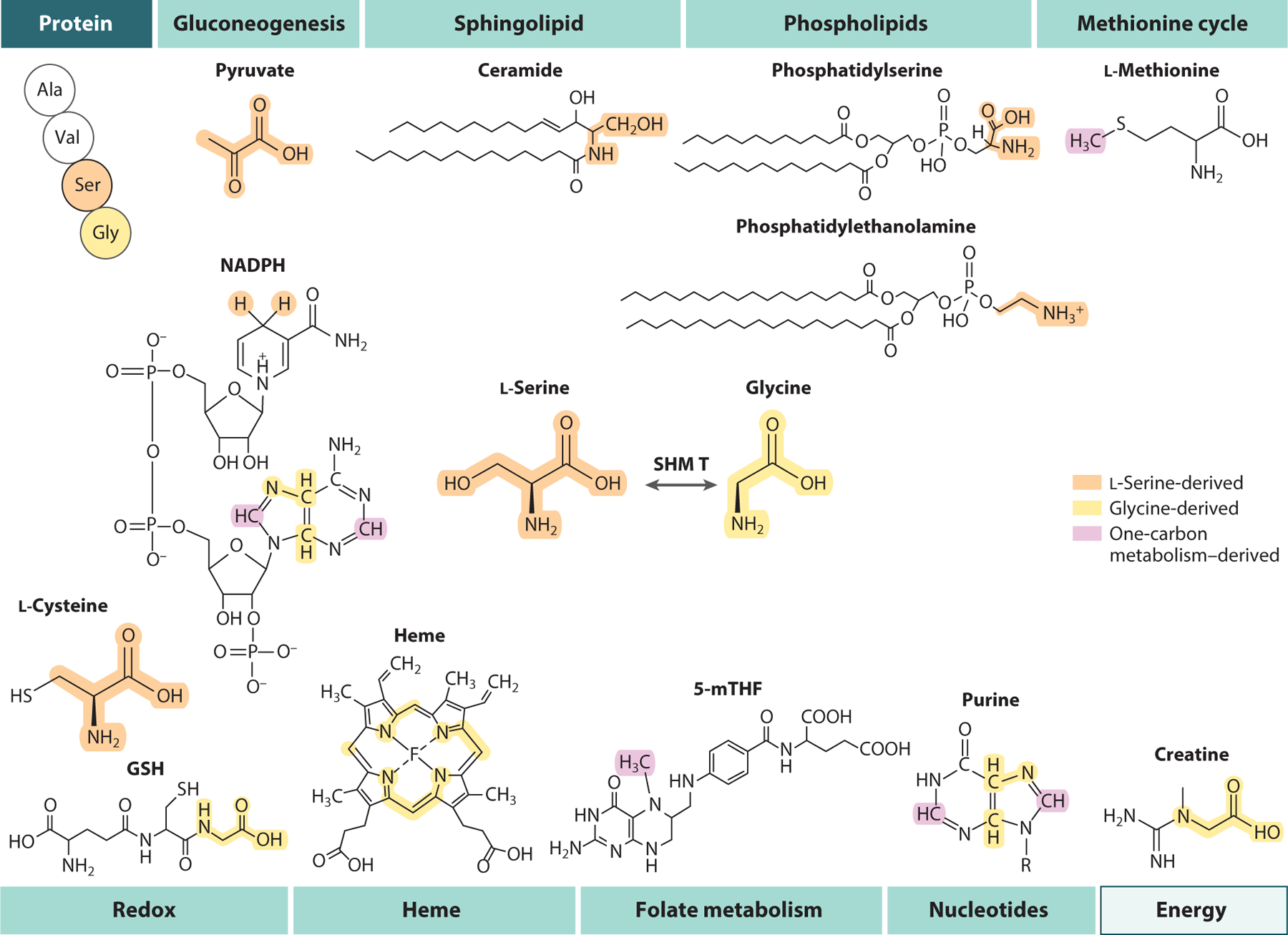
l-Serine and glycine fuel numerous metabolic pathways. l-Serine is a substrate for pyruvate biosynthesis and provides a headgroup in the synthesis of complex lipids. Via one-carbon metabolism, l-serine contributes to the methionine cycle and purine biosynthesis. Finally, through its rapid interconversion to glycine, l-serine may influence GSH biosynthesis, intracellular oxygen homeostasis (heme), nucleotide production, and mitochondrial energy metabolism (creatine). Abbreviations: GSH, glutathione; SHMT, serine hydroxymethyltransferase; 5-mTHF, 5-methyltetrahydrofolate.

Beyond serving as a precursor for protein synthesis, l-serine directly supports the generation of pyruvate for gluconeogenesis, biosynthesis of lipid headgroups (sphingolipids, phosphatidylserine, phosphatidylethanolamine), and production of the neurotransmitter D-serine (Figure 3). Via a methylcobalamin-dependent 5-methyltetrahydrofolate-homocysteine methyltransferase reaction, l-serine-derived one-carbon units can also support l-methionine regeneration and methylation, further demonstrating the diverse metabolic pathways that are dependent on l-serine availability in the body. Furthermore, l-serine contributes to the production of l-cysteine and glutathione (GSH) in the transsulfuration pathway. The activity of methylene tetrahydrofolate reductases (MTHFRs) can also serve to regenerate NAD(P)H cofactors within the mitochondria and cytosol, although the directionality of these reactions is likely to be cell and context dependent (63). As noted above, l-serine is readily converted to one-carbon units and glycine to support synthesis of thymidylate and purines, heme, and creatine, all of which are critical for cell growth, redox homeostasis, and mitochondrial function (56, 69, 117). As such, it is not surprising that altered l-serine homeostasis has been linked to numerous phenotypes in animal models (35, 36, 60, 69).

**Figure 4:**
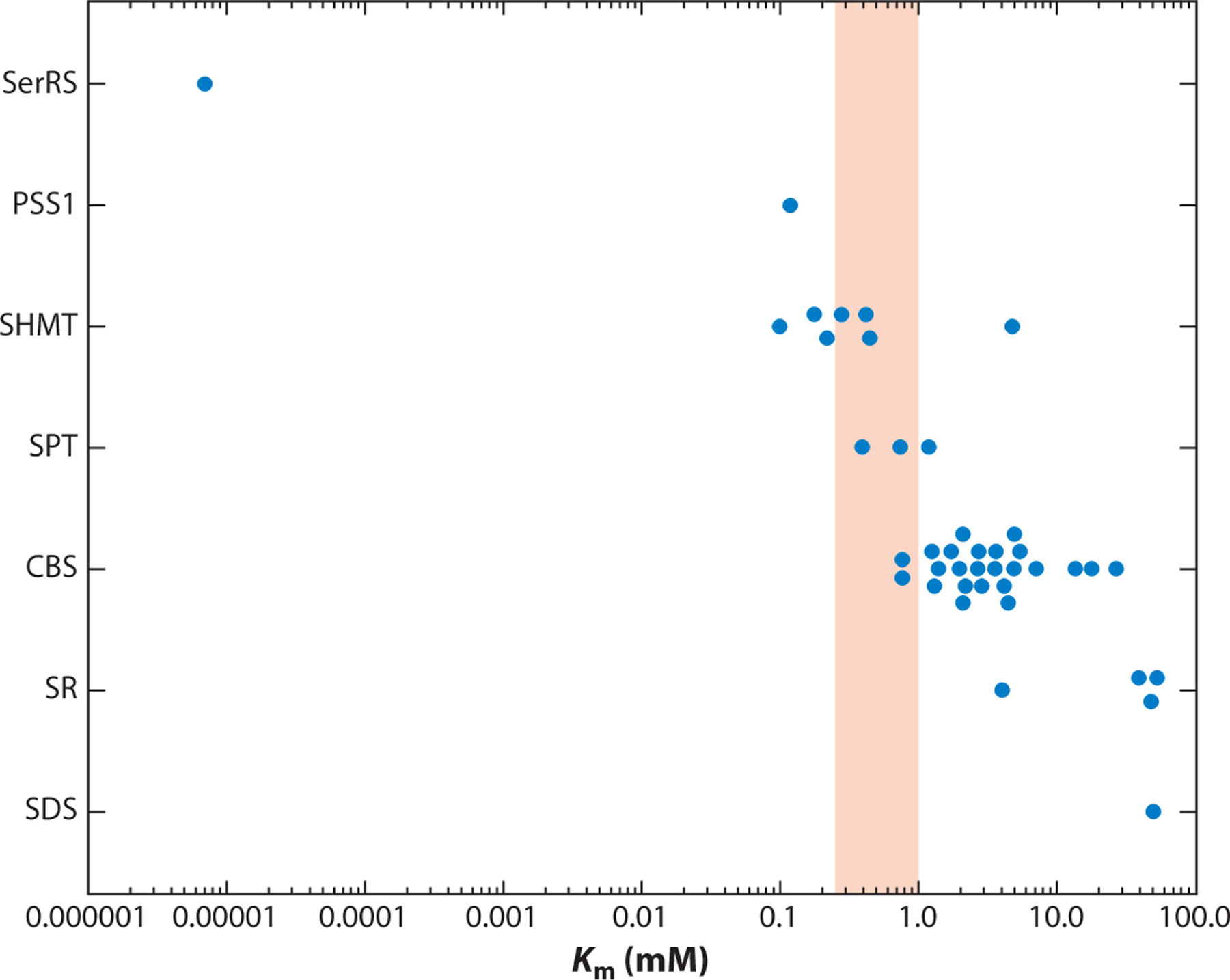
*K*_m_ values as a readout on the fates of l-serine. The *K*_m_ values for different l-serine-utilizing enzymes are depicted. The shaded area indicates the concentration of l-serine observed in xenografted tumors in mice fed either l-serine/glycine-replete or -deficient diets. Abbreviations: CBS, cystathionine B synthase; PSS1, phosphatidylserine synthase 1; SDS, serine dehydratase; SerRS, seryl-tRNA synthetase; SHMT, serine hydroxymethyltransferase; SPT, serine palmitoyltransferase; SR, serine racemase. Figure adapted with permission from Reference 80 using the BRENDA database (https://www.brenda-enzymes.org; 11).

However, a key question facing researchers is which downstream enzyme(s) specifically is influenced by physiologically low l-serine levels (see the sidebar titled Tracking the Impact of Metabolic Deficiencies Through Enzyme Kinetics). To address this question, we examined annotated values in the BRENDA database (11) of the l-serine *K*_m_ for different serine-utilizing enzymes, comparing the values with concentrations observed in tumors from mice fed serine-replete or serine-deficient diets (48). These data (Figure 4) reveal striking differences across different enzymes. For example, it is likely that tRNA synthetases are rarely limited for l-serine given the reported *K*_m_ value; however, several seryl-tRNA synthetase enzymes that exhibit distinct functionalities and codon usage in genes have been identified, suggesting that some of these enzymes may be more or less sensitive to l-serine availability (7, 61). Other enzymes with *K*_m_ values estimated to be lower than or close to that of physiological l-serine include phosphatidylserine synthase 1 and SHMT. Meanwhile, the *K*_m_ values for serine palmitoyltransferase (SPT), CBS, serine racemase, and serine dehydratase (SDS) suggest that they may be more sensitive to fluctuations in l-serine levels. While many of these enzymes are highly tissue-specific, we observed that SPT is very sensitive to dietary serine restriction in mice by exploiting the inherent promiscuity of the enzyme and the production of l-alanine-derived 1-deoxysphingolipids (see the sidebar titled Enzyme Promiscuity in Amino Acid Metabolism and Its Relation to Disease). The responsiveness of serine racemase to dietary serine supplementation has also been noted in a mouse model of Alzheimer’s disease (AD) (60), further supporting the distinct dependencies of these enzymes on serine availability.

## 3. TRANSPORT OF L-SERINE INTO CELLS

**Figure 5:**
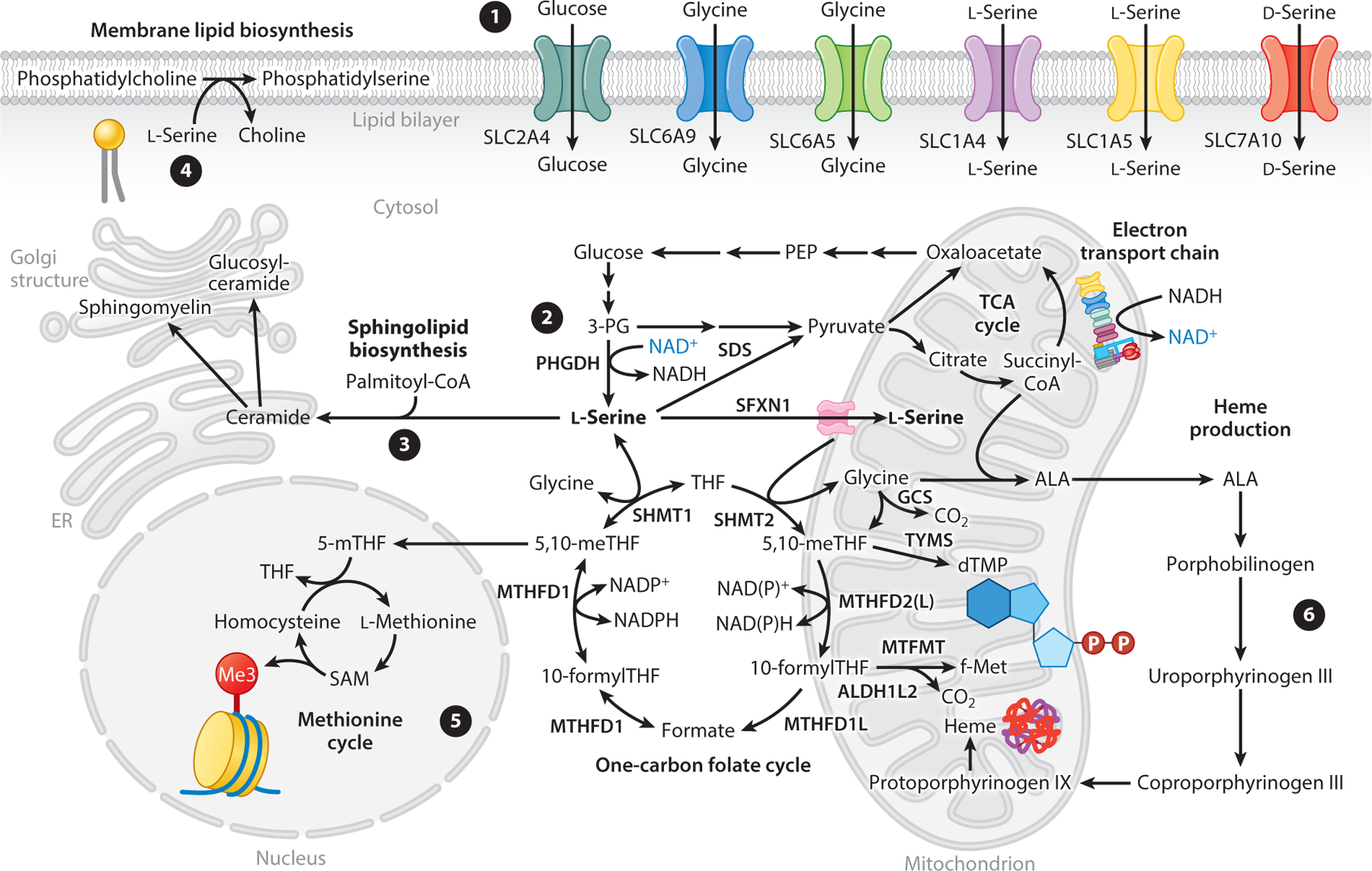
Schematic and summary of l-serine transport systems into and within cells. (①) l-Serine and D-serine are transported into cells via selective transporters. (②) Additionally, l-serine can be synthesized de novo from glucose via PHGDH. (③) SPT condenses l-serine with palmitoyl-CoA to synthesize sphingolipids including ceramides, glucosyl-ceramides, and sphingomyelin. (④) l-Serine also affects phospholipid composition, thus influencing membrane fluidity. (⑤) Via folate-mediated one-carbon metabolism, l-serine is a source of one-carbon units that remethylate homocysteine and influence posttranslational modifications and epigenetics. (⑥) Mitochondrial l-serine availability can affect glycine synthesis and thus modulate biosynthesis of heme and mitochondrial electron transport chain proteins. Abbreviations: ALA, δ-aminolevulinic acid; CDP, cytidine diphosphate; GCS, glycine cleavage system; PEP, phosphoenolpyruvate; PHGDH, 3-phosphoglycerate dehydrogenase; SAM, *S*-adenosylmethionine; SDS, serine dehydratase; SPT, serine palmitoyltransferase; TCA, tricarboxylic acid; 3-PG, 3-phosphoglycerate; 5,10-meTHF, 5,10-methylene tetrahydrofolate; 5-mTHF, 5-methyltetrahydrofolate.

Another critical step in l-serine physiology is its transport into cells across the plasma membrane or into distinct organelles. As is the case for many solute carriers, and in light of the structural similarity between l-serine and other amino acids (Figure 1), l-serine transporters are relatively nonselective, although the expression of distinct solute carriers appears to be cell specific (Figure 5). Expression and functional studies in *Xenopus* oocytes of SLC1A4 (also known as ASCT1) have demonstrated that this transporter mediates the high-affinity (*K*_m_ ∼ 30–90 μM) uptake of l-alanine, l-serine, and l-cysteine as well as a lower-affinity (*K*_m_ ∼ 140–390 μM) uptake of l-threonine in a sodium-dependent manner (3, 114). Expression of ASCT1 is ubiquitous in mammals, with the highest levels observed in brain, skeletal muscle, and pancreas (3). Indeed, genetically engineered mice deficient in ASCT1, but not SLC1A5/ASCT2, exhibited reduced levels of l-serine, D-serine, l-alanine, l-threonine, and glycine in the brain relative to wild-type controls (55). Consistent with the functional role of l-serine in the brain, ASCT1 knockout mice displayed delayed righting response and grip strength (55), implying that impaired brain l-serine uptake is not compensated for by other metabolic pathways to maintain cerebral l-serine homeostasis and leads to neuromuscular defects. On the other hand, Northern blot experiments demonstrated that ASCT2 is expressed mainly in the kidney, large intestine, lung, skeletal muscle, testis, and adipose tissue (114). Interestingly, ASCT2 is overexpressed in different types of cancer (32), and RNA interference–mediated knockdown of ASCT2 reduced mTOR signaling in proliferating cancer cells (87).

In addition to l-serine transport, ubiquitous expression of a high-affinity sodium-independent D-serine transporter (SLC7A10) has been reported (81). In contrast, in mice, Slc7a10 expression is constrained to brain, lung, placenta, and small intestine (33). Of course, any defect in l-serine transport could be compensated for by other isoforms or glycine transporters (e.g., SLC5A9, SLC6A5) combined with SHMT activity.

As noted above, l-serine metabolism is highly active in mitochondria, yet the transporters for many amino acids into mitochondria have yet to be functionally characterized. Kory et al. (57) cultured Jurkat and K562 cells in media deficient in l-serine and observed altered expression of SFXN1; they also found that SFXN1-null cells manifested a proliferation defect in the absence of l-serine, suggesting that it plays a role in serine transport. By observing labeling changes downstream of [2,3,3-^2^H]serine, these authors demonstrated a functional role for SFXN1 in l-serine transport into mitochondria (57). These results highlight key transporters involved in l-serine and D-serine homeostasis but also suggest that there is much to learn about how l-serine and glycine transport are regulated in distinct cell types and/or disease states.

## 4. SOURCES AND SINKS OF L-SERINE IN MAJOR ORGANS

As described above, cells and tissues may use diverse pathways to maintain l-serine homeostasis, including biosynthesis from glucose, regeneration from glycine, uptake and transport from the diet, or salvage from protein breakdown. In the absence of exogenous l-serine, cultured breast cancer cell lines commonly upregulate PHGDH expression, though PSAT1 protein levels vary markedly between basal and luminal cell lines (14). Consistent with these distinctions, basal cancer cells exhibit markedly higher de novo l-serine biosynthesis from glucose relative to luminal cells (14, 69). On the other hand, some human pancreatic ductal adenocarcinoma cell types exhibit low expression of PHGDH even in the absence of serine, rendering them sensitive to l-serine withdrawal in culture or dietary l-serine restriction in orthotopic xenograft models (7). Aside from cancer cells and xenograft systems, various tissues contribute to l-serine homeostasis and may act as physiological sources or sinks that influence circulating l-serine and/or local concentrations as well as disease states.

### 4.1. Kidney

Using quantitative mass spectrometry and arteriovenous differences in metabolite fluxes across different organs in mammals, Jang et al. (53) revealed that kidney and liver are net l-serine producers into the circulation. Consistent with these observations, earlier arteriovenous differences in amino acid levels in rats showed that kidney consumes a significant amount of glycine and secretes l-serine into the circulation (67), suggesting that the kidney, a major site of FOCM, may use glycine to produce l-serine for peripheral tissues.

To test this hypothesis directly, Pitts et al. (91) administered radiolabeled l-serine precursors to rats and observed that glycine accounts for a significant source of carbon in l-serine. Furthermore, feeding mice a glycine-enriched diet was associated with nearly 100% synthesis of l-serine in the canine kidney (92). Consistent with these tracer studies, the activity of PHGDH in the mammalian kidney was shown to be lower in comparison to liver, lung, or intestine (103), indicating limited l-serine biosynthesis from glucose within the kidney.

### 4.2. Liver

Hepatic l-serine synthesis has been detected from radiolabeled glucose across multiple vertebrate species, including rat, chicken, turtle, frog, and eel; hepatic PHGDH activity was highest in reptiles and lowest in fish (43). Interestingly, liver activity of SHMT, which synthesizes l-serine from glycine, was highest in mammals and lowest in reptiles (43), indicating different capacities for l-serine synthesis from glucose and glycine across vertebrate species. Using primary lamb hepatocytes, Narkewicz et al. (83) demonstrated that ∼50% of newly synthesized l-serine was derived from glycine and that primary lamb hepatocytes secreted l-serine into the media. Consistent with this observation, maximal activity of PHGDH was found to be more than 50-fold lower than that of SHMT in adult rat liver (103), suggesting that glycine is a major source of carbon for l-serine biosynthesis in liver (as is the case in kidney). Remarkably, embryonic and postnatal development has been implicated in the regulation of l-serine metabolism (83). For instance, hepatic activities of PSAT and SHMT were approximately twofold higher and lower, respectively, in fetal liver relative to the adult organ (91), suggesting that fetal liver uses distinct sources of l-serine and that postnatal development prioritizes glycine over glucose for l-serine synthesis. Consistent with primary hepatocyte data, a quantitative mass spectrometry analysis of arteriovenous differences of metabolite uptake and secretion in pigs revealed hepatic l-serine secretion (53).

The liver may also be a critical organ for l-serine catabolism and disposal. Oral gavage of [U-^13^C]serine in fasted mice indicated that l-serine was readily converted to glycine as well as pyruvate and glucose, with glycine and glucose enrichment being nearly the same at 15 min post-gavage (44). These findings indicate that l-serine may serve as a gluconeogenic precursor in mouse liver, though some contribution from renal gluconeogenesis is likely as well.

### 4.3. Brain

**Figure 6:**
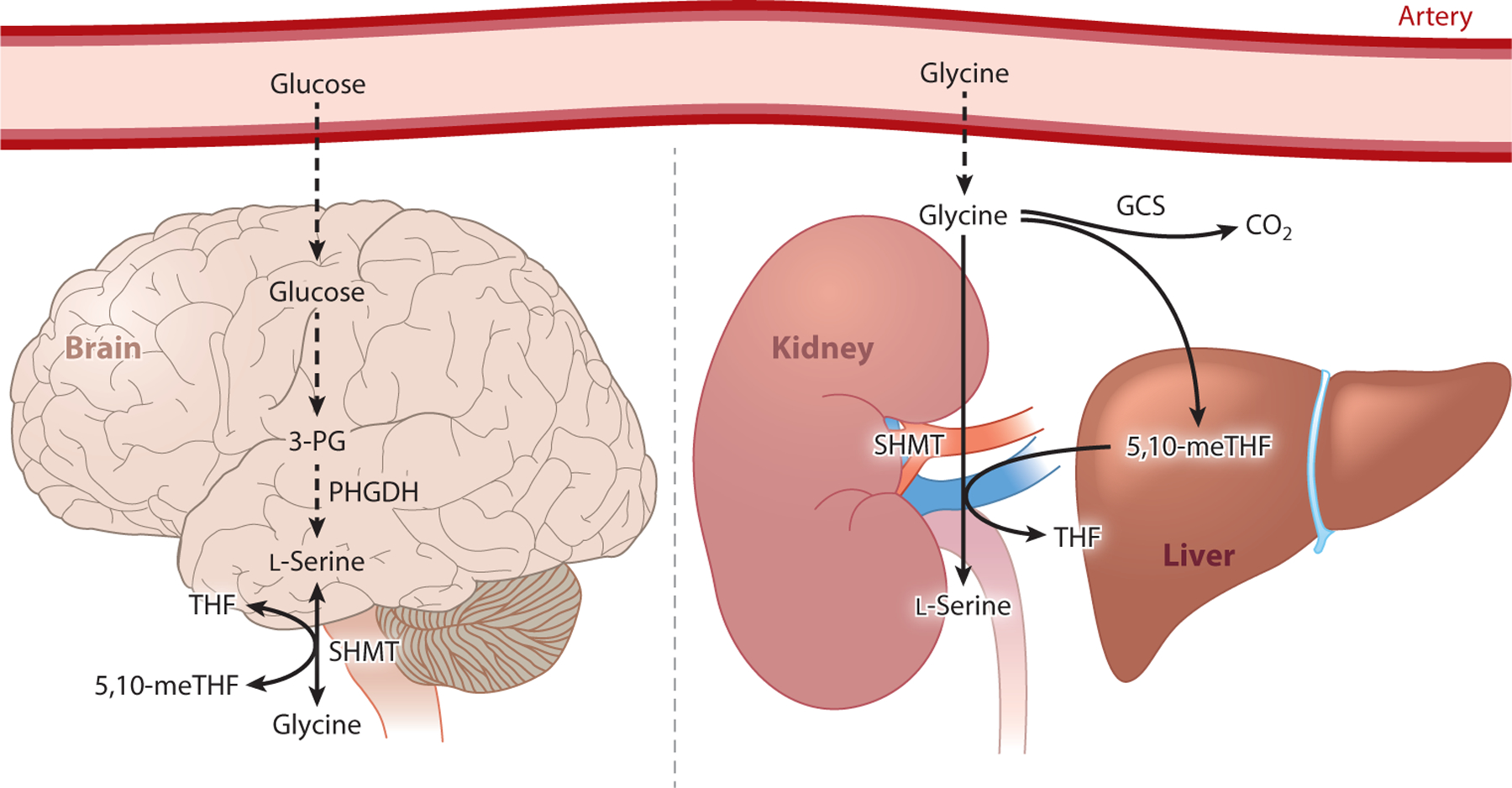
Organ-specific sources of de novo l-serine biosynthesis. Stable isotope tracers and arteriovenous metabolite differences across organs demonstrate that different organs utilize different substrates for l-serine synthesis. Astrocytes express high levels of PHGDH and rely on glucose, an important substrate for brain metabolism, to synthesize l-serine. In contrast, liver and kidney utilize predominantly glycine to synthesize and release l-serine into the circulation. Abbreviations: GCS, glycine cleavage system; PHGDH, 3-phosphoglycerate dehydrogenase; SHMT, serine hydroxymethyltransferase; 3-PG, 3-phosphoglycerate; 5,10-meTHF, 5,10-methylene tetrahydrofolate.

l-Serine is essential for brain development and function. l-Serine is actively synthesized and secreted by cultured astrocytes for provision to neurons, and supplementation of culture media with 100 μM l-serine improves neuronal survival and neurite growth (75). PHGDH expression in the adult mammalian brain is restricted to astrocytes in gray and white matter (120), correlating with the more glycolytic phenotype of astrocytes versus neurons. Furthermore, embryonic deletion of PHGDH in mice leads to hypoplasia of the telencephalon, diencephalon, mesencephalon, olfactory bulbs, ganglionic eminence, and cerebellum (121). Quantification of lipid classes in wild-type littermates and PHGDH knockout mice indicated that, relative to control littermates, the PHGDH knockout mice exhibited significant reductions in different lipid classes, including phosphatidylserine and sphingolipids (121). These findings suggest that PHGDH-mediated l-serine biosynthesis is indispensable for lipid biosynthesis and brain development. The importance of local serine synthesis by PHGDH in the brain is further emphasized by the low blood–brain barrier permeability of l-serine (44, 62). In support of this notion, deficiencies in the de novo l-serine biosynthesis pathway in human individuals highlight its critical role in brain development and function (as discussed in Section 5). D-Serine is a neurotransmitter produced by serine racemase from l-serine, which actively occurs in the brain. This path may serve as a sink or outlet for l-serine synthesis under some conditions, and D-serine levels are responsive to dietary serine supplementation in mice (60). However, more detailed studies are required to understand how D-serine metabolism influences l-serine physiology. Collectively, these findings demonstrate that the central nervous system and peripheral tissues (i.e., circulation driven by liver and kidney) rely on distinct metabolic pathways for l-serine biosynthesis (Figure 6).

### 4.4. Eye

The mammalian eye is a complex organ composed of distinct, spatially organized cell types. As in the brain, serine synthesis is limited in retinal neurons (i.e., rods and cones) and occurs predominantly in supporting cells, including Müller glia and retinal pigmented epithelia (RPE). The retina requires significant biosynthetic capacities for proteins and lipids to support organelle turnover and lipid homeostasis (23). Consistent with findings in astrocytes and oligodendrocytes in the brain, both Müller glia and RPE exhibit high PHGDH expression and de novo l-serine biosynthesis, as measured by [U-^13^C]glucose tracing (26, 122). Likewise, as discussed below, aberrant l-serine biosynthesis in the eye contributes to genetically driven macular disease through its effect on sphingolipids and other metabolic processes (26, 35).

### 4.5. Heart

Under baseline conditions, the mammalian heart relies primarily on fatty acid oxidation (66). Using arteriovenous difference in metabolite uptake/release, Murashige et al. (76) showed that while skeletal muscle consumes l-serine from the circulation, the heart releases l-serine into the bloodstream in heathy human participants. A growing body of evidence suggests that significant metabolic remodeling occurs in the failing heart (85) and includes increased utilization of carbohydrates and ketone bodies with concomitant suppression of fatty acid oxidation. Indeed, elevated one-carbon metabolism has recently been observed in patient responders with improved myocardial structure and function following left ventricular assist device–induced mechanical unloading (5). Furthermore, improved contractile function was observed in induced pluripotent stem cell–derived cardiomyocytes (iPSC-CMs) from patients with genetic dilated cardiomyopathy (DCM) upon treatments with drugs that activated the l-serine biosynthetic network (90). Interestingly, in addition to normalized glucose and fatty acid metabolism, improved contractile function in patient-derived iPSC-CMs was associated with increased expression of genes related to l-serine and glycine metabolism (90). Interestingly, l-serine synthesis as well as glucose oxidation were altered in DCM iPSC-CMs compared with cells that were genetically corrected, and serine supplementation also drove improvements in iPSC-CM function. These findings highlight roles for l-serine and one-carbon metabolism in the failing heart, but the mechanisms driving these phenomena remain unclear.

## 5. INBORN ERRORS OF L-SERINE METABOLISM

Although rare, inborn errors of l-serine metabolism have provided insights into the clinical outcomes associated with impaired l-serine biosynthesis, with symptoms predominantly affecting neurological function and growth.

### 5.1. Neu–Laxova Syndrome

Neu–Laxova syndrome, first described in 1971, is clinically associated with microcephaly, anomalies of the limbs, atrophic gyri, and absence of the corpus callosum, along with atrophy of the cerebrum, cerebellum, and pons (84). An autozygome analysis of affected patients revealed missense variant mutations in *PHGDH*, linking de novo l-serine metabolism with cognitive and motor function (102). In line with these genetic defects, a mass spectrometry–based quantitative analysis of dried blood samples from patients with Neu–Laxova syndrome revealed significantly reduced levels of l-serine and glycine (113), further supporting the notion of aberrant l-serine metabolism as a contributing factor to Neu–Laxova syndrome.

### 5.2. PHGDH Deficiency

First described in 1996, PHGDH deficiency presents a rather well-defined spectrum of clinical phenotypes, including severe neurodevelopmental disorder with microcephaly, seizures, and psychomotor retardation (52, 116). Although the onset and development of the disease can vary from infancy to adulthood, in most patients symptoms are evident before birth, with congenital microcephaly and intrauterine growth retardation (116). Biochemical characteristics of PHGDH-deficient patients include low concentrations of l-serine in cerebrospinal fluid (CSF) and low to borderline concentrations of l-serine and glycine in plasma (116). While the diagnostic utility of amino acids in urine for PHGDH deficiency patients remains unclear (116), PHGDH activity in patient-derived skin fibroblasts exhibited only 12–25% activity relative to heathy controls (108). Considering the low l-serine levels in CSF and plasma, dietary l-serine supplementation has been tested as a therapeutic approach, with dietary l-serine supplementation ranging from 80 to 700 mg/(kg·day) reported to improve biochemical and clinical outcomes in these patients (116).

### 5.3. PSAT Deficiency

A 2007 study reported two cases of PSAT deficiency (45). The first patient exhibited feeding difficulties, jerky movements, seizures, and an arrest of head circumference. Brain magnetic resonance imaging analysis revealed hypoplasia of cerebellar vermis and pons as well as multiple white matter abnormalities with no overt signs of hypomyelination. Consistent with other inborn errors of l-serine metabolism, the concentration of l-serine was significantly reduced in CSF and plasma. Although amino acid therapy corrected systemic l-serine deficiency, it did not improve clinical outcomes, consistent with the importance of in situ biosynthesis within the brain. Interestingly, neurological defects in a younger sister diagnosed with PSAT deficiency within 24 h after birth were reversed with an immediate l-serine treatment, suggesting a therapeutic window of opportunity after birth to normalize l-serine homeostasis and prevent and/or reverse accumulating neurological dysfunction.

### 5.4. PSPH Deficiency

PSPH deficiency was reported in 1997 in a boy with Williams–Beuren syndrome (WBS) (116). In addition to typical WBS clinical features, the patient displayed intrauterine growth defects, feeding difficulties, hypospadias, and microcephaly, all of which are rare complications of WBS. Quantification of l-serine revealed reduced levels in plasma and CSF, and therapeutic administration of l-serine normalized CSF amino acid levels and modestly improved progress of head circumference growth.

## 6. ABERRANT L-SERINE METABOLISM IN NEUROLOGICAL DISORDERS

Consistent with the striking neurological phenotypes of patients with inborn errors of l-serine metabolism discussed above, l-serine has emerged as a potential driver of various neuroretinal disorders. In turn, these findings have spurred interest in investigating whether dietary l-serine supplementation can improve outcomes in these cases.

### 6.1. Macular Telangiectasia Type 2

Macular telangiectasia type 2 (MacTel) is a rare, familial disease affecting visual acuity and macular function, with onset of symptoms typically occurring at ∼40 years of age but in some cases much earlier (i.e., twenties). Genome-wide association studies in MacTel patients identified significant loci near genes associated with l-serine, glycine, and FOCM, including *PHGDH*, *PSPH*, *ALDH1L1*, and *CPS1*; strikingly, a serum metabolomics analysis identified l-serine and glycine as the most differentially abundant metabolites in circulation (100). A separate collapsing analysis of exome sequences from MacTel patients identified PHGDH haploinsufficiency as the most significantly enriched genetic change in this cohort, with numerous deleterious coding variants identified and confirmed via enzyme assay (26). Altered levels of nonessential amino acids were confirmed to occur in MacTel patients, with reduced l-serine and elevated l-alanine observed relative to controls in a more targeted analysis of patient sera (35, 100). Furthermore, mimicking systemic l-serine deficiency by feeding mice with serine/glycine-free diets impaired photopic b-waves and led to thermal hypoalgesia, suggesting that prolonged l-serine deficiency leads to retinopathy and peripheral neuropathy.

Notably, circulating l-serine correlated negatively with noncanonical sphingolipids (e.g., hydrolyzed 1-deoxysphinganine) in MacTel patients (35). This finding suggests that aberrant l-serine and glycine homeostasis may link with sphingolipid metabolism via SPT promiscuity (see the sidebar titled Enzyme Promiscuity in Amino Acid Metabolism and Its Relation to Disease), consistent with earlier in vitro reports (28). Indeed, dietary l-serine restriction in mice was also associated with an altered lipid profile in various tissues, including RPE and choroid; the most striking change was accumulation of noncanonical 1-deoxysphingolipids (35). These atypical sphingolipids build up in the context of gain-of-function mutations in SPT subunits (*SPTLC1* or *SPTLC2*) that are present in patients with the neurological disorder hereditary sensory and autonomic neuropathy type 1 (HSAN1) (27, 89). Patients with this ultrarare disorder experience sensory (depressed reflexes, altered pain and temperature perception) and autonomic dysfunction (gastroesophageal reflux, postural hypotension, excessive sweating) (4) and have been clinically characterized with progressive degeneration of dorsal root ganglion and motor neurons, distal sensory loss, distal muscle wasting and weakness, and variable neural deafness (30, 50). Although HSAN1 is not directly linked to low l-serine levels (and patients have higher circulating l-serine compared with controls; 41), supplementation of l-serine is expected to reduce 1-deoxysphingolipid synthesis. One year of dietary l-serine supplementation to HSAN1 patients improved clinical outcomes on the Charcot–Marie–Tooth neuropathy score, version 2, relative to a placebo-treated group, and attenuated circulating levels of 1-deoxysphingolipids (36). This finding demonstrates that dietary l-serine supplementation can override genetically induced SPT promiscuity to restore sphingolipid diversity and clinical outcomes in HSAN1 patients.

Strikingly, a visual examination of available HSAN1 patients demonstrated that most could also be diagnosed with MacTel, in particular, some cases with early-onset macular and visual defects (35). Although most MacTel patients do not harbor SPT subunit mutations, circulating l-serine and l-alanine levels correlated strongly with 1-deoxysphinganine from patient blood, providing evidence that low l-serine can drive the accumulation of neurotoxic 1-deoxysphingolipids. While the accumulation of 1-deoxysphingolipids is likely insufficient to drive MacTel, these species may serve as a robust longitudinal biomarker of lipid alterations in patients with low l-serine levels. Furthermore, these findings support the potential for l-serine supplementation to normalize circulating amino acid levels and potentially slow the progression of visual defects.

### 6.2. Diabetic Peripheral Neuropathy

**Figure 7:**
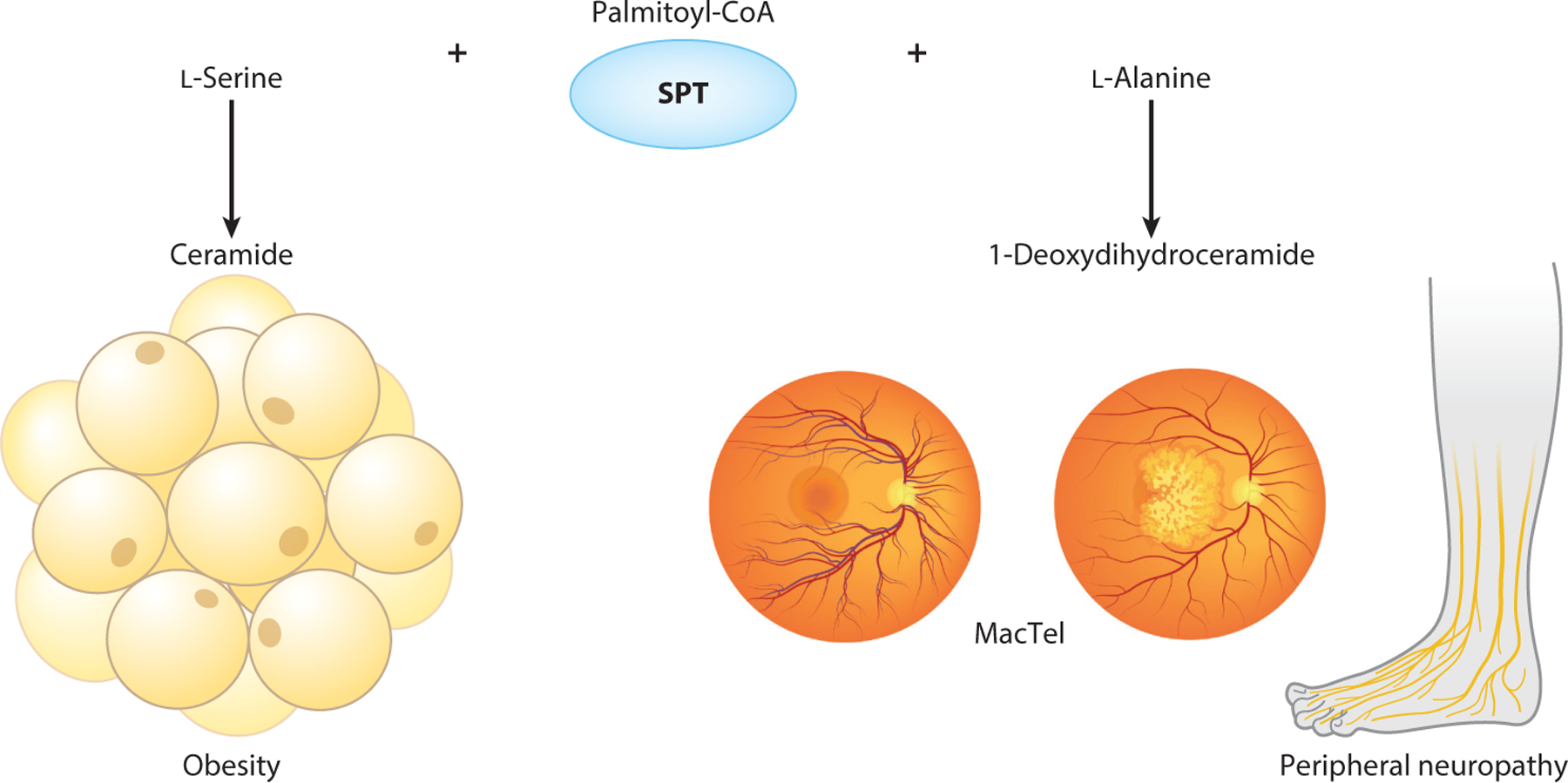
Schematic of SPT promiscuity and its roles in obesity and peripheral neuropathy. Metabolically and phenotypically, SPT promiscuity is linked to aberrant sphingolipid diversity in the context of metabolic disorders, where it manifests an increased synthesis of canonical ceramides that are implicated in the development of obesity and insulin resistance, as well as noncanonical 1-deoxydihydroceramides involved in small nerve fiber loss and MacTel-associated retinopathy. Abbreviations: MacTel, macular telangiectasia type 2; SPT, serine palmitoyltransferase. Figure adapted from images created with BioRender.com.

Diabetes is associated with various comorbidities, including peripheral neuropathy, particularly in severe cases. Although peripheral neuropathy is commonly thought to be driven by hyperglycemia and redox stress, recent studies have shown correlations between low l-serine levels and indicators of peripheral neuropathy. For instance, a comparison of patients with different degrees of obesity found that circulating levels of l-serine were lowest in obese patients with neuropathy, while plasma l-serine levels correlated negatively with 1-deoxydihydroceramides (31). These findings therefore implicate l-serine homeostasis in lipid remodeling and peripheral nerve dysfunction. Consistent with this clinical observation, a prolonged (6-month) dietary l-serine/glycine restriction in combination with a high-fat diet in C57BL/6 mice accelerated development of peripheral neuropathy, as evidenced by thermal hypoalgesia and loss of small nerve fibers (44). At the same time, l-serine restriction attenuated high-fat diet–induced obesity (Figure 7). In support of this model, dietary l-serine supplementation (10% l-serine diet) into streptozotocin-treated type 1 diabetic rats mitigated the accumulation of deoxysphingolipids and improved indicators of diabetic peripheral neuropathy such as tactile sensation and nerve conduction velocity (88). Likewise, dietary l-serine supplementation decelerated the kinetics of thermal and tactile hypoalgesia in BKS-*db/db* mice (44, 119). Furthermore, dietary l-serine supplementation to streptozotocin-treated mice reduced damage to pancreatic acinar cells and lowered markers of pancreatitis (13), suggesting that therapeutic administration of l-serine mitigates pancreatic injury. As such, the mechanisms through which l-serine ameliorates diabetic peripheral neuropathy remain somewhat unclear and may include reduction of 1-deoxysphingolipids or improvement of insulin secretion and/or sensitivity. However, l-serine supplementation does not reduce hyperglycemia in BKS-*db/db* models despite the improvement in neuropathy phenotypes, suggesting that some forms of diabetic peripheral neuropathy are more directly driven by l-serine deficiency as opposed to hyperglycemia.

### 6.3. Alzheimer’s Disease

Aberrant l-serine homeostasis has emerged a potential modifier of AD. Although protein expression of PHGDH in brain samples in AD was shown to be increased or decreased (60, 71), a quantitative mass spectrometry analysis demonstrated reduced levels of l-serine and glycine in brain samples from AD patients (71), suggesting that elevated levels of PHGDH may signal regional l-serine deficiency. Consistent with a clinical analysis of l-serine levels in AD patients, a mouse model of AD also exhibited reduced l-serine availability in the hippocampus associated with synaptic deficits (60). Moreover, adeno-associated virus (AAV)-mediated PHGDH inactivation in the brain decreased tissue l-serine and glycine levels and impaired synaptic plasticity and spatial memory (60), demonstrating that reduced l-serine levels in the brain contribute to cognitive dysfunction. Importantly, dietary l-serine supplementation normalized l-serine and D-serine levels in this AAV-mediated PHGDH-inactivated model of AD and restored synaptic plasticity and spatial memory. Collectively, these data imply that hippocampal l-serine deficiency contributes to neuronal dysfunction associated with AD and that dietary l-serine supplementation restores hippocampal levels of l-serine and improves cognitive function. Intriguingly, a specific form of AD-like neurodegeneration emerged on the island of Guam in the twentieth century, and this disease has been suggested to be caused by aberrant incorporation of β-methylamino-l-alanine, a nonprotein amino acid produced by cyanobacteria, in place of l-serine within the proteome (77, 78). This hypothesis has generated additional interest in employing l-serine supplementation to mitigate neurodegeneration in these contexts (60; see ClinicalTrials.gov identifier NCT03062449).

## 7. L-SERINE HOMEOSTASIS AND CHRONIC DISEASES

The disease states and comorbidities described above link l-serine deficiency to various neuroretinal defects. Notably, both MacTel and AD show positive associations with type 2 diabetes (15, 100), a chronic disease that alters systemic metabolism via insulin resistance, which is likely to affect key tissues important for l-serine homeostasis (e.g., liver and kidney). Therefore, chronic diseases that manifest low circulating l-serine and glycine availability may drive comorbidities that share characteristics with MacTel, HSAN1, or AD. Metabolic insights described below suggest how and why these disease states result in low circulating l-serine and offer promise for the therapeutic benefits of supplementation.

### 7.1. Diabetes and Obesity

**Figure 8:**
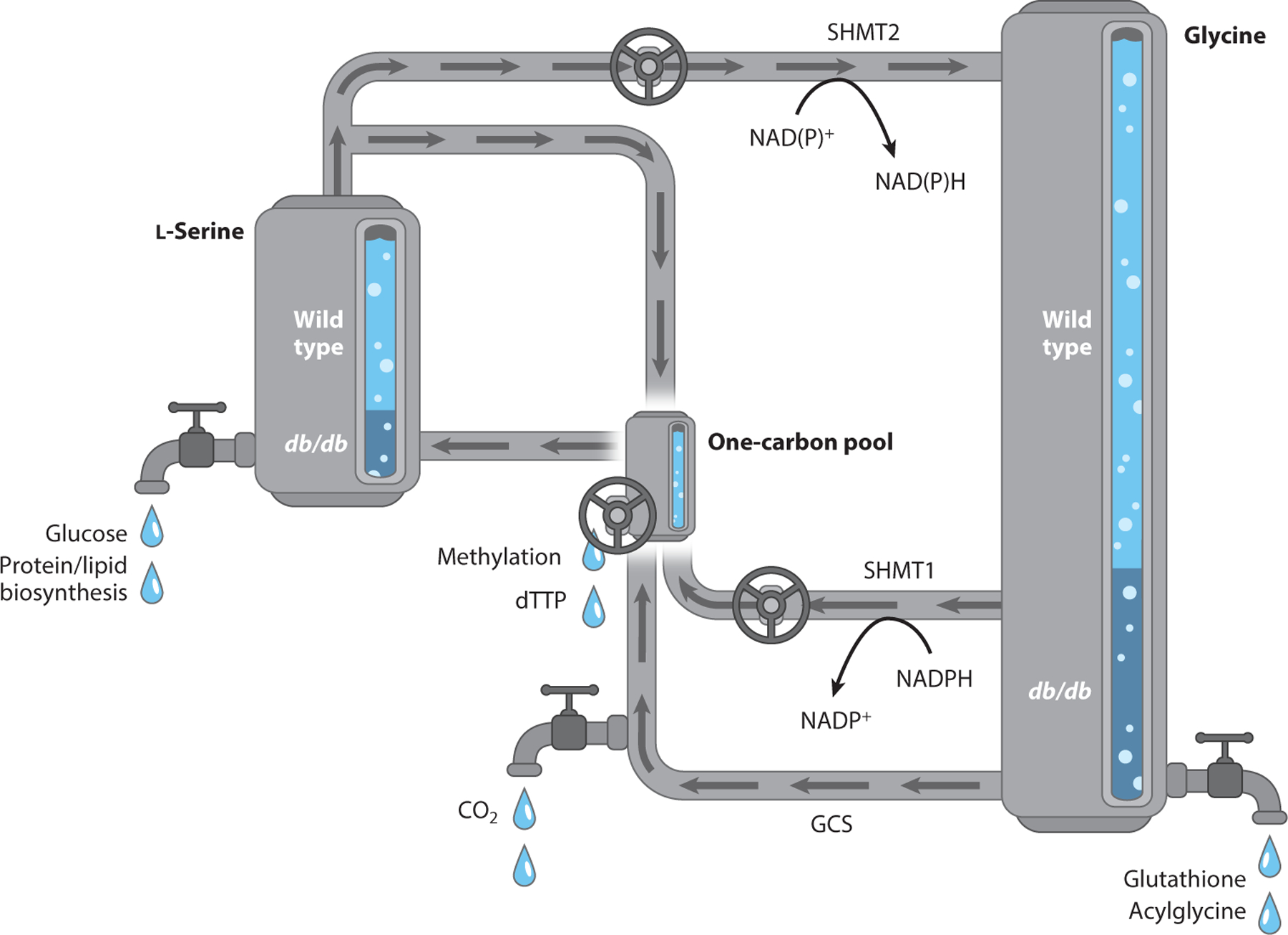
Sources and sinks of l-serine in diabetes. l-Serine and glycine metabolic pools within the liver are depicted as tanks, scaled to match the molar ratio to volume (the one-carbon pool is not to scale and is significantly smaller in moles). Metabolic disorders, including diabetes, are associated with aberrant l-serine and glycine metabolism, which may arise as a result of altered one-carbon interconversions or dysregulated disposal to various pathways that are depicted as system outlets. Relative abundances of l-serine and glycine in diabetic mouse liver (*db*/*db*) versus wild-type mouse liver are depicted. Abbreviations: GCS, glycine cleavage system; SHMT, serine hydroxymethyltransferase.

Although classically linked with glucose intolerance and dyslipidemia, diabetes and obesity have been associated with aberrant systemic essential and nonessential amino acid homeostasis. For example, using high-pressure liquid chromatography, Felig et al. (29) revealed reduced levels of l-serine and glycine in patients with diabetic ketoacidosis and obesity, indicating that metabolic diseases are linked with systemic reductions in l-serine and glycine levels. This metabolic fingerprint has recently been recapitulated with mass spectrometry–based metabolomics in patients with type 1 and type 2 diabetes (8, 86, 110), validating earlier observations. Using BKS-*db/db* mice, we observed significantly reduced levels of l-serine in liver and kidney relative to control mice (44). These data suggest that reduced hepatic and renal l-serine output (or increased catabolism) may contribute to systemically reduced l-serine levels. Using animal models of aberrant insulin and glucagon signaling, including prolonged (48 h) fasting and alloxan-induced type 1 diabetes, Sallach et al. (99) demonstrated that fasting and diabetic mice exhibited elevated liver serine dehydratase (SDS) activity and reduced PHGDH activity, indicating increased capacity for l-serine disposal and reduced capacity for de novo l-serine biosynthesis. Increased SDS and decreased PHGDH transcription occurs in diabetic mice, which may contribute to altered l-serine homeostasis (44). Likewise, transcript and protein expression of glycine decarboxylase, a key component of the glycine cleavage system that breaks down glycine, is upregulated in a mouse model of type 2 diabetes (*db/db*) and in response to fasting (54). Together, these data imply that aberrant insulin and/or glucagon signaling impairs capacities to synthesize l-serine and glycine and elevates routes of disposal of these two amino acids. Figure 8 depicts this concept as biochemical reactors.

Consistent with the model in which insulinopenia or hyperglucagonemia contributes to systemic l-serine disposal, glucagon infusion into neonatal and adult human individuals reduced circulating levels of l-serine and glycine (64, 74, 94). Additionally, deletion of the glucagon receptor resulted in elevated levels of l-serine and glycine (34), suggesting that hyperactive glucagon signaling in diabetes and obesity likely contributes to the low systemic l-serine and glycine observed in such patients.

To better gauge what drives reduced levels of l-serine (and glycine) in diabetes, we implemented a serine tolerance test (STT) to assess whole-body l-serine homeostasis (44). Oral administration of l-serine and glucose to mice at doses of 400 mg/kg and 2 g/kg, respectively, drives an insulin response in healthy, fasted mice which is compromised in diabetes models (see the sidebar titled Amino Acid Tolerance Tests: Looking Beyond Steady State). Application of the STT to streptozotocin-treated mice (type 1 diabetes model) and BKS-*db/db* mice (type 2 diabetes model) demonstrated that diabetic animals exhibit a reduced l-serine area under curve (AUC_SER_) compared with healthy controls, in contrast to AUC_GLUC_, which is elevated in diabetic mice (as expected). These results further support the notion that aberrant insulin and glucagon signaling result in increased clearance and/or reduced absorption of l-serine to lower circulating levels in the animal, despite the higher amount administered to heavier diabetic animals. These findings suggest that l-serine supplementation may be more challenging to achieve in diabetic subjects but also highlight a potential approach for diagnosing patients who are susceptible to serine-associated peripheral neuropathy.

### 7.2. Chronic Kidney Disease

Stable isotope tracers and quantitative metabolomics studies have demonstrated that the kidneys utilize circulating glycine to synthesize l-serine that is then released into the systemic circulation (53, 67, 115). Interestingly, a quantitative analysis of circulating l-serine and glycine revealed decreased levels of plasma l-serine and elevated levels of glycine (10) as a signature of early-stage renal failure (creatine clearance of 33 mL/min). This observation is consistent with findings in an animal model of chronically uremic rats in which circulating plasma l-serine was significantly reduced relative to control animals (107). Another clinical study quantified the dynamics of kidney amino acid metabolism in patients with renal insufficiency and observed that l-serine secretion from kidneys was reduced by 80% to 90% (111). Collectively, these findings may suggest that chronic renal failure leads to impaired renal glycine uptake, which in turn contributes to the decreased l-serine levels in the circulation observed in patients. This observation highlights a clear contribution of kidney dysfunction to the metabolic fingerprint of patients with chronic kidney disease (CKD). Interestingly, while l-serine homeostasis is altered in these settings, elevation of circulating D-serine levels has emerged as a sensitive biomarker for CKD progression. Specifically, circulating D-serine concentration was elevated in CKD patients (47), and incubation of human kidney 2 (HK-2) cells with D-serine induced cell cycle arrest at the G_2_/M phase, further implicating D-serine accumulation in the development of kidney disease.

### 7.3. Liver Disease

A quantitative mass spectrometry analysis of healthy controls and patients with nonalcoholic steatohepatitis (NASH) revealed that, consistent with other metabolic diseases, circulating levels of l-serine are significantly reduced in such patients (9, 19). Interestingly, hyperinsulinemic–euglycemic clamp performed in control subjects and NASH patients demonstrated that the insulin-mediated decrease in circulating l-serine and glycine was blunted in NASH patients, suggesting that insulin resistance alters l-serine and glycine homeostasis (9). Furthermore, a clinical study investigating the impact of NASH on whole-body glycine disposal found that NASH patients exhibited elevated glycine flux toward serine, as evidenced by the l-serine-labeling pattern with [U-^13^C]glycine (19). This result indicates that reduced levels of l-serine and glycine in patients may be driven by upregulated l-serine disposal in diseased patients.

An analysis of human and mouse liver samples with nonalcoholic fatty liver disease revealed decreased expression of genes synthesizing glycine, identifying another mechanism that may contribute to decreased systemic and hepatic glycine levels (97). In another study using gene expression analysis from human liver biopsy samples, Mardinoglu et al. (72) identified glycine as a limiting factor for GSH biosynthesis. Testing the hypothesis that dietary supplementation of l-serine, *N*-acetyl-l-cysteine (NAC), and nicotinamide riboside (NR) to mice fed with a Western diet may influence the development of NASH, Mardinoglu et al. (73) found that a cocktail of dietary l-serine, NAC, and NR reduced hepatic triglyceride content. Consistent with the rodent model, dietary l-serine supplementation at 200 mg/(kg·day) for 14 days in human patients with NASH revealed reduced circulating levels of markers of liver disease, alanine aminotransferase and aspartate aminotransferase, as well as attenuated hepatic fat content, as shown by magnetic resonance spectroscopy (73). Interestingly, dietary supplementation with glycine or glycine-containing tripeptide (DT-109) also reduced circulating glucose, lipids, transaminases, proinflammatory cytokines, and steatohepatitis in mice with established NASH induced by a diet high in fat, cholesterol, and fructose (97). Mechanistically, dietary glycine or DT-109 supplementation stimulated de novo GSH synthesis, linking reduced glycine availability in liver disease with antioxidant capacity (97). Alternatively, modulating FOCM has been suggested to alter liver-specific NADPH production to directly influence de novo fatty acid synthesis, providing other mechanisms by which targeting l-serine metabolism may affect lipid metabolism in tissues exposed to prolonged lipid overload (123).

Collectively, these findings highlight distinct chronic disease states related to the liver and kidney that influence systemic l-serine metabolism in human patients, resulting in reduced levels of l-serine and glycine that, when ameliorated with dietary supplements, have the potential to improve disease phenotypes.

### 7.4. Cancer

Tumor cells require excessive nutrients to support their growth and survival, and genes within the l-serine synthesis and FOCM pathways are commonly expressed at higher levels in cancer cells (103). Later, analyses of public oncogenomic data (93) revealed that *PHGDH* is genomically amplified in triple-negative breast cancer and melanoma (65, 93). Consistent with these observations, ectopic overexpression of PHGDH in mice bearing mutant *Braf* expression in melanocytes enhanced tumor growth (106). More recent studies, however, have demonstrated that cancer sensitivity to PHGDH may differ between primary breast tumors and breast cancer–derived lung metastases, revealing greater complexity in the exploitation of l-serine metabolism as a metabolic vulnerability (95). Taken together, these studies demonstrate that PHGDH-mediated de novo l-serine biosynthesis is positively associated with tumor growth and that targeting PHGDH via genetic or pharmacological inhibition may reduce cancer development and progression, at least in some contexts.

Given the negative impact of a reduced de novo l-serine biosynthesis pathway on tumor growth, modulating l-serine availability has become an attractive therapeutic approach to reduce cancer progression. Removal of l-serine or of l-serine and glycine from the media in HCT116, A549, RKO, and SW480 cells demonstrated their dependency on exogenous l-serine availability, an effect that was potentiated by p53 mutation (59, 69). In line with in vitro experiments, feeding of tumor-bearing mice with diets deficient in l-serine and glycine reduced tumor growth (69, 80) and enhanced the efficacy of chemotherapies administered in conjunction with l-serine-restricted diets (70). Similar findings were obtained in human pancreatic ductal adenocarcinoma xenografts that were incapable of inducing PHGDH expression (7). Together, these data demonstrate that genetic or dietary impairment in de novo l-serine biosynthesis or l-serine availability delays cancer growth in tissue culture and in vivo settings.

l-Serine fuels numerous biochemical pathways. While l-serine restriction in cultured cells has dramatic effects on the metabolome, the impact of l-serine-deficient diets is more muted given the physiological buffering afforded by different tissues. However, these dietary regimens result in decreased nucleotide pools as well as the accumulation of 1-deoxysphingolipids within the tumor (80). Importantly, diminishing accumulation of deoxysphingolipids in tumor-bearing mice with low-dose myriocin during feeding with a l-serine/glycine-free diet accelerated tumor growth, providing an alternative mechanism by which dietary l-serine/glycine starvation may affect tumor growth. How could elevated levels of noncanonical sphingolipids affect tumor growth? While the mechanism(s) is likely to be complex, use of a mutant SPT construct caused an accumulation of deoxysphingolipids that compromised endocytosis, a mechanism by which cells import nutrients necessary for proliferation and expansion (16). On the other hand, a recent report highlighted that silencing of PHGDH may act to increase metastasis, potentially by altering the balance between l-serine and sialic acid synthesis (98), further demonstrating the contextual dependence of these metabolic pathways.

### 7.5. Inflammation

The metabolic demands of immune cells are very high, and adequate l-serine availability is necessary for immune cell function. Considering the high proliferative rate of immune system cells, l-serine metabolism has been implicated in the regulation of T cell function. For instance, activated T cells increase expression of enzymes in the de novo l-serine biosynthetic pathway (68), and both murine and human T cells show significant de novo l-serine biosynthesis from glucose (68). Consistent with high expression of l-serine synthesizing genes in T cells, dietary l-serine restriction reduces T cell response following *Listeria monocytogenes* infection, supporting the idea that l-serine plays an essential role in the regulation of T cell function and expansion. Mechanistically, [U-^13^C]serine tracing revealed its contribution to nucleotide biosynthesis, which is critical for T cell expansion, reflecting bioenergetic and proliferative demands.

Consistent with this study (68), another investigation reported impaired lipopolysaccharide (LPS)-induced macrophage IL-1β mRNA expression in cells cultured in the absence of l-serine (96). Stable isotope tracing experiments showed a significant contribution of l-serine-derived carbon into nucleotide, *S*-adenosylmethionine, and GSH biosynthesis, suggesting that l-serine contributes to energy balance, methylation, and redox biology of activated macrophages. Importantly, the in vivo use of a PHGDH inhibitor in mice reduced IL-1β secretion in response to LPS challenge and improved survival. On the other hand, in LPS-stimulated macrophages, *Il6* and *Tnfa* expression was further elevated in the absence of media l-serine and glycine, whereas *Il10* expression was decreased relative to full media (58). Collectively, these data suggest that during infection l-serine availability orchestrates macrophage inflammatory response and survival.

### 7.6. Muscle Stem Cells and Regeneration

After injury, muscle stem cells become activated, proliferate, and differentiate to regenerate functional tissue. However, evidence suggests that muscle progenitors have a somewhat limited capacity for de novo l-serine biosynthesis (37). Restriction of dietary l-serine and glycine reduces the expression of markers associated with stem cell proliferation and limits muscle regeneration (37). Human muscle progenitor cells growing in the absence of l-serine and glycine exhibit reduced GSH abundance and elevated reactive oxygen species (ROS), whereas treatment with cell-permeable GSH improves ROS and cell proliferation. Interestingly, the same study observed reduced levels of skeletal muscle l-serine in aged subjects, a finding that may imply that age-associated reductions in skeletal muscle l-serine contribute to impaired muscle regeneration and frailty (37).

Collectively, these results highlight the diverse sources and sinks of l-serine across different cell types, organs, and disease states. As occurs in most critical metabolic pathways, inherent biochemical redundancies enhance the resilience of organisms to nutritional stresses. l-Serine is a prime example, given the many biochemical pathways to which it is linked.

## 8. QUANTIFYING WHOLE-BODY L-SERINE METABOLISM

The above findings highlight two critical points: (*a*) circulating l-serine and glycine levels, their turnover, and their biomarkers vary in the context of disease states and across the human population (35), and (*b*) low l-serine levels drive specific disease comorbidities through their impact on different cell types and biochemical pathways. Does this mean that l-serine may sometimes be considered a conditionally essential amino acid? Potentially, in selected cases or patients (see the sidebar titled Essential Versus Nonessential: Nutrition or Metabolism?). However, by and large, most people take in and retain adequate l-serine for survival. Given the importance of endogenous metabolism in sustaining l-serine (and glycine) levels in circulation, quantifying l-serine turnover in patients may be valuable for identifying those patients who may be responsive to serine supplementation or other therapies.

**Figure 9:**
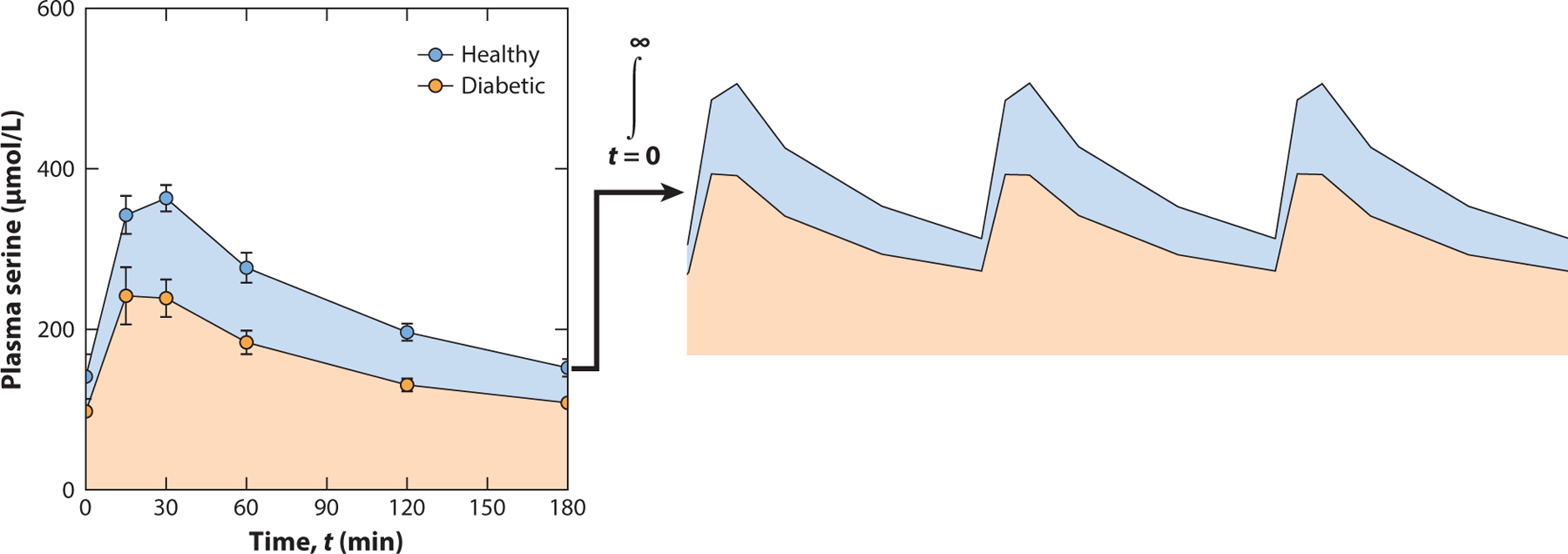
A serine tolerance test (STT) as a potential diagnostic tool to gauge serine and one-carbon homeostasis. An STT enables quantitation of serine metabolism in response to a challenge, capturing elements of absorption, retention, and catabolism. Although exacerbated in the context of an STT, minor differences that consistently occur in diseased patients, when integrated over time, may accumulate to drive disease pathologies. This concept may be a fundamental tenet of chronic diseases, in contrast to the more severe disease states arising from inborn errors of l-serine metabolism. Figure adapted from Reference 44 (CC BY 4.0).

An STT enables quantitation of l-serine metabolism in response to a challenge, capturing elements of absorption, retention, and catabolism (44). The administration of an STT challenges the system and exacerbates changes that may not be detectable via steady-state metabolomics or other methods (see the sidebar titled Amino Acid Tolerance Tests: Looking Beyond Steady State). Even minor fluctuations that occur repeatedly during feeding/fasting cycles, integrated over time, can cause downstream biochemical changes (e.g., 1-deoxysphingolipids) to accumulate over time and ultimately reach a threshold at which they cause disease. Modeling postprandial metabolism in this manner highlights another important concept, since feeding drives insulin signaling cascades that prioritize biosynthetic fluxes during this time to ensure adequate nutrients are available to fuel downstream pathways (e.g., protein, nucleotide, and lipid biosynthesis). As shown in Figure 9, diabetic mice receive a higher amount of l-serine and glucose (given their higher weight) but experience a lower peak concentration and AUC_SER_ during this postprandial time—which is the most critical for lipid synthesis. Notably, this reduced AUC_SER_ contrasts with the increased AUC_GLUC_ observed in a classical glucose tolerance test in diabetics, demonstrating the striking dysregulation of l-serine homeostasis in diabetic mouse models. While clinical studies are required to appreciate these differences in human settings, recent data correlating low circulating l-serine and high 1-deoxysphingolipids in diabetic peripheral neuropathy patients suggest that these differences may be relevant in human disease states (31).

## 9. FUTURE DIRECTIONS

In this article, we have reviewed the diverse sources and sinks of l-serine present in mammals, including diet, synthesis from glucose, regeneration from glycine, biosynthesis, and catabolism to glucose, highlighting the key organs involved in the maintenance of l-serine homeostasis. We have also described the pleiotropic fates of l-serine, which contribute to many downstream pathways important for cell function. Key insights are obtained in considering patients with inborn errors in l-serine metabolism as well as their phenotypes and responsiveness to dietary supplements. We have also described several diseases and comorbidities that have recently been associated with low circulating l-serine, including macular, cognitive, and peripheral sensory impairment. Key challenges that remain include gaining a fundamental understanding of (*a*) the molecular mechanisms that drive these diverse phenotypes, (*b*) why chronic disease states ultimately drive low l-serine levels, (*c*) how these findings can be therapeutically exploited, and (*d*) how we can potentially diagnose patients susceptible to l-serine-associated pathologies.

## ENZYME PROMISCUITY IN AMINO ACID METABOLISM AND ITS RELATION TO DISEASE

Some essential and nonessential amino acids, including glycine, l-cysteine, l-alanine, and l-threonine, share structural similarities with l-serine. Prolonged aberrant amino acid homeostasis may therefore lead to differential substrate usage owing to minor differences in their chemical potential. For example, serine palmitoyltransferase (SPT) condenses l-serine with palmitoyl-CoA to synthesize sphingolipids including ceramides. When l-serine availability is limited, or in sensory neuropathy patients harboring gain-of-function variants in *SPTLC1* or *SPTLC2*, SPT incorporates other amino acids, including l-alanine, glycine, and l-threonine, to produce noncanonical sphingolipids that cannot be further metabolized to complex sphingolipids or degraded via phosphorylation. Promiscuous activation of tRNA synthetases caused by editing defects or substrate availability between l-serine, l-alanine, and noncanonical amino acids may also contribute to neurodegeneration in some contexts.

## TRACKING THE IMPACT OF METABOLIC DEFICIENCIES THROUGH ENZYME KINETICS

Given the diverse metabolic fates of amino acids and the distinct metabolic demands of cells and tissues throughout the body, it is difficult to estimate which pathways may become limited for a given analyte. One approach to estimate where the so-called fluxome may be most sensitive to specific metabolic deficiencies is to examine and compare enzyme–substrate affinities for different amino acid–utilizing enzymes. Although annotated databases are often incomplete and in vitro measurements may not be representative of actual physiological affinities, a direct comparison between enzyme expression and *K*_m_ values can direct one to the most affected pathways for a given tissue and substrate deficiency.

## AMINO ACID TOLERANCE TESTS: LOOKING BEYOND STEADY STATE

Metabolism and biology become most interesting when they are challenged. Engineers characterize the resilience of physical systems and software by performing a load test. The same approach is effectively used in biological systems and human patients; for example, the oral glucose tolerance test is widely used to assess the insulin sensitivity of patients in the clinic. In biochemical terms, such challenges better mimic the dynamic nature of feeding and capture the activity of enzymes that are responsive to insulin or have higher *K*_m_ values which may be dysregulated in the context of disease. More standard approaches, such as metabolomics or steady-state isotope infusions, fail to capture all these details and may not be representative of the dynamic physiological changes which occur during feeding. Thus, a serine tolerance test (STT) may be most effective in assessing whole-body l-serine homeostasis.

## ESSENTIAL VERSUS NONESSENTIAL: NUTRITION OR METABOLISM?

When teaching human physiology and biochemistry, we broadly classify amino acids as being essential or nonessential. These classifications have specific dietary meanings regarding nutritional intake needs but can be misleading when applied to “nonessential” amino acids that might be considered less important or impactful. In fact, nonessential amino acids such as l-serine or glycine may become chronically low due to changes in endogenous metabolic pathways, regardless of intake, rendering them conditionally essential in some contexts.

## SUMMARY POINTS

1. l-Serine, a nonessential amino acid important for numerous biochemical processes, can be derived from the diet or synthesized de novo from glucose or glycine.
2. In mammals, l-serine synthesis is organ specific, with the brain relying on glucose and the kidney and liver utilizing glycine.
3. Patients with mutations in the l-serine synthesis genes display marked neurological deficits.
4. Acquired l-serine deficiency associated with macular telangiectasia or diabetes presents with small sensory nerve fiber neuropathies.
5. Aberrant l-serine homeostasis may be diagnosed in patients with a serine tolerance test (STT).

## FUTURE ISSUES

1. What are the mechanistic bases of reduced systemic l-serine and glycine in patients and animal models with metabolic disorders?
2. Does elevated whole-body l-serine disposal precede the development of sensory neuropathy in patients with diabetes?
3. Can l-serine supplementation or targeted drugs attenuate the development of sensory neuropathy in patients?
